# Intimate partner violence and maternal health services utilization: evidence from 36 National Household Surveys

**DOI:** 10.1186/s12889-021-10447-y

**Published:** 2021-02-25

**Authors:** Jessica Leight, Nicholas Wilson

**Affiliations:** 1 (IFPRI) Poverty, Health and Nutrition Division, 1201 Eye St., Washington, D.C 20005 USA; 2 Department of Economics, Reed College, 3203 SE Woodstock Blvd, Portland, OR 97202 USA

**Keywords:** Intimate partner violence, Maternal health, Antenatal care, Skilled delivery care, Postnatal care

## Abstract

**Background:**

High rates of maternal mortality and intimate partner violence (IPV) are both major worldwide health challenges. Evidence from single-country samples suggests that IPV may be an important risk factor for low utilization of maternal health services, but there is little large-scale evidence on this association. This paper evaluates whether IPV is a risk factor for low utilization of maternal health services in a large cross-country sample, and also compiles evidence on the relative effects of different forms of IPV.

**Methods:**

We analyze the association between intimate partner violence and utilization of maternal health care, using a dataset compiling all Demographic and Health Surveys that report data on intimate partner violence. Using data on 166,685 women observed in 36 countries between 2005 and 2016, we estimate logistic regression models to analyze the relationship between lifetime experience of IPV and utilization of antenatal care (ANC), facility delivery care, and postnatal care. We estimate both unadjusted models and models adjusted for geographic and sociodemographic characteristics that are generally correlated with utilization of maternal health care (including age, education, number of children, wealth status, marital status, and urbanity).

**Results:**

Lifetime experience of any IPV is associated with decreased use of maternal health services in a broad sample of births observed in lower and middle-income countries: in particular, the utilization of four or more ANC visits, the number of ANC visits, and the utilization of facility care at birth. This association remains statistically significant even after adjusting for country of residence, subnational region of residence, and additional individual-level covariates; however, there is no statistically significant association between experience of any IPV and postnatal care. The only form of IPV significantly associated with care utilization is physical IPV.

**Conclusions:**

Women experiencing physical intimate partner violence show lower levels of utilization of maternal health services in a large sample of developing and middle-income countries. Given that reduced utilization of maternal health services is correlated with maternal and neonatal health outcomes, this pattern suggests that IPV prevention may be an important component of interventions targeting enhanced maternal and neonatal health.

## Background

Maternal mortality is a major worldwide public health challenge. The World Health Organization estimates that every day in 2017, approximately 810 women died due to preventable causes linked to pregnancy and childbirth [1]. High rates of maternal mortality reflect in part low utilization rates of high-quality reproductive health care: the majority of maternal deaths are preventable if women receive access to high-quality antenatal, delivery and postnatal care provided by a skilled professional [1].

A variety of factors can lead to observed low levels of utilization of maternal health services, including limited service accessibility and quality, socioeconomic barriers to accessing available services, and limited information about the availability of services or their value [2, 3]. However, recent literature has also increasingly analyzed the role of psychosocial barriers in accessing reproductive health care, particularly in developing countries, and one key barrier in many contexts is the prevalence of intimate partner violence [4].

Intimate partner violence is a worldwide challenge, and globally, 30% of women experience physical and/or sexual violence by an intimate partner (IPV) in their lifetime [5]. IPV has both immediate and long-term adverse health, social and economic consequences for women and their families [6, 7]. In addition, IPV adversely affects reproductive and sexual health [8–11] through a range of pathways, including its links with socioeconomic status, empowerment, and broader dimensions of physical and mental health [12]. One particularly important pathway that will be the focus of this analysis is the reduced utilization of reproductive health care.

In the existing literature, a number of papers have analysed the relationship between IPV and utilization of reproductive care in specific contexts in Latin America, sub-Saharan Africa, and South Asia [13–25]. The existing papers use a wide variety of data sources, analyse a variety of indicators of care utilization (including measures linked to utilization of antenatal care, delivery care and other forms of reproductive care), focus on different measures of IPV, and have highly variable sample sizes. While a recent systematic review aggregated data from seven published studies and concluded IPV has a negative relationship with utilization of antenatal care and delivery care, even this analysis included data from only 11 countries [4].

The objective of this paper is to provide a comprehensive analysis of the association between intimate partner violence and utilization of maternal health care, using a dataset compiling all Demographic and Health Surveys that report intimate partner violence and also meet certain minimal additional inclusion criteria, as specified subsequently in the methods section. This allows us to compile data on 166,685 women observed in 36 countries between 2005 and 2016, all of whom responded to a standardized set of questions around exposure to intimate partner violence as well as utilization of antenatal, delivery and postnatal care during their most recent pregnancy. Accordingly, we provide an exhaustive, worldwide analysis of whether IPV is a risk factor for low utilization of maternal health services.

## Methods

The DHS are a set of national household surveys conducted in low-income and middle-income countries; this description of the surveys draws substantially on Wilson (2019) [26]. The primary sampling procedure in the surveys is a stratified two-stage cluster design, in which clusters are sampled in proportion to population size using national census records, and households are sampled with equal probability from a list of households in the cluster. Within each household, at least one female age 15—49 is surveyed.

For this analysis, we draw on the sample of DHS surveys that collect data on lifetime intimate partner violence from a subset of females included in the full survey. (Women who are currently married / partnered or who are divorced, separated or widowed are eligible for the IPV module, while never-married women are not eligible; if there is more than one woman eligible in a given household, the respondent is randomly selected.) For those countries that have multiple DHS rounds available including IPV data, we draw on the most recent survey at the time of sample construction. Note we exclude some country surveys in which questions are posed only about violence experienced from non-partners (as opposed to violence experienced from intimate partners); we also exclude surveys in which incomplete data about intimate partner violence was collected. For example, the IPV module in Bangladesh did not include questions about emotional violence, and the module in Pakistan did not include questions about sexual violence; both are excluded.

Within this sample of country-level surveys, we further restrict our sample to women who report a live birth within the last five years and thus report utilization of maternal health care. (Women who have never experienced a live birth, or whose last live birth falls outside this window, did not report any information about their utilization of maternal health care, and accordingly cannot be included in this analysis.) We also restrict our study sample to respondents with complete information for the full set of health services outcomes and covariates. Following these sample restrictions, any individual country survey that has fewer than 500 observations meeting these criteria is dropped (thus excluding Azerbaijan, São Tomé and Príncipe, and the Ukraine). Imposing these conditions yields a sample of 166,685 women.

### Outcome variables: health care utilization

In order to analyze the utilization of reproductive health care, we construct five variables. Each female respondent reporting a live birth in the last five years reports this information for her most recent birth. The first variable is a dichotomous variable equal to one if the respondent reports attendance at antenatal care from any medically trained provider. The second is a dichotomous variable equal to one if the respondent reports attendance at four or more antenatal care visits, consistent with the recommendations for antenatal care provided by the WHO [27]. The third variable is a continuous variable equal to the number of antenatal care visits reported (characterized by a minimum of zero and a maximum of 18).

The fourth variable is a dichotomous variable equal to one if the respondent reports delivery of the child in a health facility. The fifth variable is a dichotomous variable equal to one if the respondent reports attendance at postnatal care from any medically trained provider within three days of the delivery. WHO guidelines now recommend four postnatal visits for all new mothers, of which the first two are recommended within 72 h [28]. Our measure therefore captures this dimension of short-term utilization of postnatal care.

### Explanatory variables: intimate partner violence

We construct four measures of intimate partner violence using the standard DHS data. Our strategy follows that employed in the DHS reports, as well as the definitions laid out by the World Health Organization in its landmark multicountry study on violence against women [29]. Our strategy is also consistent with the definitions of IPV used in single-country analyses to date of the association between IPV and maternal health care utilization [4].

More specifically, we construct four dichotomous variables. A respondent is identified as experiencing emotional violence during her lifetime if she responds affirmatively to any of the following four questions: at any time during her lifetime, did her intimate partner insult her or make her feel bad about herself; belittle or humiliate her in front of others; taken actions to scare or intimidate her on purpose; or threaten to hurt someone she cared about. A respondent is identified as experiencing physical violence if she respondents affirmatively to any of the following five questions: at any time during her lifetime, did her intimate partner slap her or throw something at her; push or shove her; hit her with a first or something else; kick, drag or beat her; choke or burn her; or threaten or actually use a weapon against her. A respondent is identified as experiencing sexual violence if she responds affirmatively to any of the following three questions: at any time during her lifetime, did her intimate partner physically force her to have sexual intercourse when she did not want to; did she have sexual intercourse when she did not want to because she was afraid of her partner; or was she forced to do something sexual she found degrading or humiliating.

In addition, we construct a dichotomous variable “any intimate partner violence” that is equal to one if the respondent reports experiencing any form of intimate partner violence during her lifetime.

### Covariates

In our statistical analysis, we also explore adjusting for a number of sociodemographic covariates that have been theoretically and empirically linked to both intimate partner violence and the utilization of reproductive health care [2–4]. The first set is a set of dichotomous variables for country of residence. The second set is an additional set of dichotomous variables for subnational region of residence (e.g., province) as indicated in the DHS “region” variable, and the following sociodemographic variables: dichotomous variables for the respondent’s age in years, dichotomous variables for the respondent completing primary, secondary or tertiary education, a dichotomous variable for current marital status, total number of children born, dichotomous variables for wealth quintile, and a dichotomous variable for urban residence. (The inclusion of dichotomous variables for subnational region allows the analysis to adjust for systematic geographic variation within-country in the supply or availability of maternal health services, or systematic variation in other factors shaping care utilization.)

### Statistical analysis

We first present descriptive statistics to characterize the multicountry sample utilized in the analysis. We then examine associations between intimate partner violence and utilization of reproductive health care using an unadjusted model (Model 1). For the four dichotomous variables, we estimate logistic regression models. For the continuous variable of interest (number of ANC visits), we estimate ordinary least squares regression.

We first present unadjusted models in order to identify the statistical relationship of interest without adjusting for geographic or demographic variables. In addition, we estimate two models adjusted for additional covariates. The second model adjusts for a set of dichotomous variables for country of residence (Model 2). The third model adjusts for dichotomous variables for country of residence, dichotomous variables for subnational region of residence, and the set of sociodemographic characteristics described above (Model 3). In order to examine potential heterogeneity in the effects of IPV over time, Model 3 is also estimated for three time periods: 2005—2009, 2010—2014, and 2015—2016. All models employ heteroskedasticity-robust standard errors clustered at the country level to account for within-country correlation in unobservable characteristics that may be correlated with the outcome variables of interest.

All analysis was conducted in Stata Version 16.1 MP [30]. For the dichotomous variables, we present odds ratios and associated confidence intervals. For the continuous variable, we present estimated coefficients and associated confidence intervals.

Our study used publicly available, de-identified secondary data and is accordingly deemed exempt from ethical review by the Institutional Review Board.

## Results

### Sample characteristics

Tables 1 and 2 report characteristics for the sample. In both tables, the sample is characterized by country (in alphabetical order).

**Table 1:** Characteristics of Study Populations, by Country

Country	Survey	Sample	IPV variables				Other characteristics							
			Any	Emotional	Physical	Sexual	Completed	Completed	Any	Married	Urban	Age	Total	Wealth
			IPV	IPV	IPV	IPV	primary	secondary	tertiary				children	quintile
	year	size	(%)	(%)	(%)	(%)	(%)	(%)	(%)	(%)	(%)			
Afghanistan	2015	14,070	52.3%	32.6%	48.3%	8.1%	10.3%	4.0%	1.7%	99.1%	24.7%	29.5	4.5	2.9
Burkina Faso	2010	7567	15.8%	9.9%	11.4%	1.6%	9.2%	0.5%	0.3%	97.0%	25.1%	28.9	3.8	3.0
Cambodia	2014	1696	24.6%	20.8%	14.4%	4.5%	43.6%	7.4%	3.1%	95.3%	23.8%	29.2	2.5	3.0
Cameroon	2011	2740	55.1%	38.8%	42.1%	14.6%	55.1%	5.0%	3.3%	87.6%	43.9%	27.9	3.7	2.9
Chad	2014–15	2835	29.5%	19.8%	22.6%	8.9%	12.6%	1.5%	0.6%	94.1%	20.6%	28.1	4.6	2.9
Colombia	2015–16	9398	32.8%	18.3%	27.8%	5.0%	89.0%	55.2%	28.4%	74.0%	70.1%	27.5	2.2	2.3
Comoros	2012	1314	9.9%	7.5%	5.8%	1.6%	37.9%	11.0%	7.0%	93.7%	34.7%	29.3	3.7	2.7
Cote d’Ivoire	2011–12	3716	28.4%	15.9%	23.0%	4.6%	17.2%	1.8%	0.9%	86.9%	34.7%	28.9	3.7	2.7
Republic of the Congo	2013–14	4416	55.8%	36.2%	45.5%	25.5%	44.5%	7.1%	1.5%	88.9%	27.9%	28.8	4.0	2.6
Egypt	2014	3581	29.9%	18.3%	24.5%	4.1%	79.5%	61.1%	17.5%	98.2%	42.0%	29.1	2.7	3.2
Gabon	2012	2843	47.8%	29.1%	40.5%	12.6%	64.9%	5.7%	3.6%	75.0%	61.9%	28.7	3.7	2.2
Gambia	2013	2577	29.4%	16.5%	22.5%	3.1%	29.1%	8.0%	2.5%	93.1%	40.3%	29.1	3.8	2.6
Guatemala	2014–15	3671	23.9%	20.0%	13.8%	3.6%	49.1%	12.5%	4.2%	88.6%	36.2%	28.0	2.9	2.7
Haiti	2012	4032	29.5%	22.5%	15.8%	11.5%	38.0%	3.4%	2.5%	87.8%	34.3%	29.5	3.2	2.6
Honduras	2011–12	6872	30.6%	27.2%	16.7%	4.9%	61.6%	13.2%	3.9%	83.8%	33.7%	27.7	2.8	2.5
India	2005–6	25,972	27.5%	11.2%	22.5%	7.2%	51.3%	13.6%	8.7%	98.3%	38.8%	27.2	2.8	3.1
Jordan	2012	4284	29.8%	21.6%	19.2%	7.4%	93.8%	46.5%	34.2%	99.0%	69.4%	31.7	3.7	2.6
Kenya	2014	3013	40.7%	26.6%	32.9%	10.4%	52.0%	16.7%	6.3%	85.1%	34.6%	28.7	3.5	2.6
Kyrgyz Republic	2012	2477	28.3%	10.7%	25.5%	4.2%	99.7%	90.9%	45.9%	95.7%	26.9%	29.3	2.7	2.9
Liberia	2006–07	1525	40.6%	31.6%	29.9%	9.0%	14.3%	1.4%	0.4%	82.8%	21.8%	29.5	4.0	2.3
Malawi	2015–16	3794	40.0%	26.5%	23.9%	19.0%	31.3%	7.5%	1.5%	85.8%	17.0%	27.8	3.2	2.9
Mali	2012–13	2329	44.7%	31.9%	29.8%	14.3%	12.4%	1.7%	0.9%	97.4%	27.7%	28.5	3.9	3.1
Moldova	2005	1138	24.3%	17.7%	16.9%	2.7%	99.0%	39.4%	22.8%	94.0%	55.2%	27.4	1.7	3.4
Namibia	2013	1075	21.2%	14.5%	16.1%	5.2%	74.4%	22.3%	5.5%	55.8%	45.4%	29.4	2.9	2.8
Nepal	2011–12	1512	32.7%	17.3%	23.1%	15.5%	42.7%	15.5%	6.1%	98.7%	22.0%	27.0	2.6	2.7
Nigeria	2013	15,118	25.6%	19.8%	14.9%	5.7%	50.7%	24.4%	7.2%	95.3%	34.8%	29.2	3.9	2.9
Rwanda	2015	855	34.7%	21.9%	26.4%	9.0%	37.0%	5.6%	1.8%	84.6%	21.5%	29.9	2.9	2.9
Sierra Leone	2013	2621	46.4%	25.8%	41.2%	6.4%	21.6%	3.9%	1.5%	88.9%	30.7%	29.4	3.9	2.9
Tajikistan	2012	2283	24.0%	10.6%	19.7%	3.3%	95.9%	57.4%	17.4%	97.6%	36.0%	29.4	2.9	3.3
Tanzania	2015–16	5310	43.4%	30.5%	34.5%	11.6%	67.3%	10.4%	0.6%	84.8%	25.2%	29.3	3.7	3.0
The Philippines	2013	4179	21.1%	15.1%	13.1%	5.6%	86.7%	58.9%	26.6%	93.5%	39.7%	30.2	3.1	2.5
Timor-Leste	2009–10	1579	36.7%	7.4%	34.8%	1.7%	49.3%	16.4%	1.9%	97.0%	22.6%	31.5	4.4	2.8
Togo	2013–14	3851	37.5%	31.4%	21.6%	7.8%	29.6%	2.1%	1.2%	92.6%	31.3%	30.1	3.6	2.8
Uganda	2011	1275	57.3%	40.0%	41.5%	26.3%	34.9%	5.3%	4.2%	87.2%	22.7%	28.5	4.2	2.8
Zambia	2013–14	7415	44.5%	22.7%	36.0%	16.1%	50.0%	9.2%	3.4%	82.4%	39.1%	28.8	3.9	2.7
Zimbabwe	2015	3752	43.2%	29.2%	29.6%	11.0%	87.6%	6.9%	5.8%	86.2%	38.9%	28.7	2.8	3.1
*Full sample*	2005–16	166,685	34.0%	21.3%	26.0%	8.2%	49.3%	17.8%	8.0%	91.3%	36.3%	28.7	3.4	2.8

Notes: All variables are dichotomous variables. “Any IPV” is any emotional, physical, or sexual violence by partner in lifetime. “Emotional violence” is any emotional violence by partner in lifetime. “Physical violence” is any physical violence by partner in lifetime. “Sexual violence” is any sexual violence by partner in lifetime. Physical violence does not include sexual violence. The variables capturing “completed primary”, “completed secondary”, and “completed tertiary” refer to educational attainment

**Table 2:** Maternal Health Services Utilization in Study Populations, by Country

Country	Survey	Sample	Any ANC visit	4+ ANC visits	Total ANC visits	Facility delivery	Postnatal visit
	year	size	(%)	(%)		(%)	(%)
Afghanistan	2015	14,070	56.8%	17.4%	1.7	48.1%	36.9%
Burkina Faso	2010	7567	95.7%	34.3%	3.1	75.1%	77.8%
Cambodia	2014	1696	94.0%	73.8%	5.0	82.6%	87.9%
Cameroon	2011	2740	88.4%	64.5%	4.3	66.1%	44.3%
Chad	2014–15	2835	59.6%	29.5%	2.2	21.4%	14.9%
Colombia	2015–16	9398	96.7%	89.4%	6.8	95.3%	8.5%
Comoros	2012	1314	92.1%	57.9%	4.5	76.7%	59.7%
Cote d’Ivoire	2011–12	3716	91.0%	42.6%	3.3	57.5%	76.3%
Democratic Republic of Congo	2013–14	4416	88.1%	45.3%	3.4	76.5%	43.3%
Egypt	2014	3581	90.2%	83.6%	8.7	88.4%	84.4%
Gabon	2012	2843	91.8%	68.8%	4.6	84.7%	64.9%
Gambia	2013	2577	99.5%	77.0%	4.8	62.6%	76.5%
Guatemala	2014–15	3671	96.2%	86.8%	7.1	70.1%	82.5%
Haiti	2012	4032	89.7%	66.8%	4.8	34.1%	42.2%
Honduras	2011–12	6872	96.7%	88.3%	6.4	80.0%	84.6%
India	2005–6	25,972	79.8%	44.0%	3.9	42.2%	49.1%
Jordan	2012	4284	99.3%	94.7%	8.6	99.2%	94.9%
Kenya	2014	3013	94.3%	54.7%	3.8	58.9%	58.5%
Kyrgyz Republic	2012	2477	98.2%	85.9%	6.5	99.6%	99.1%
Liberia	2006–07	1525	91.5%	59.3%	4.5	0.0%	21.8%
Malawi	2015–16	3794	98.5%	51.1%	3.7	94.0%	4.5%
Mali	2012–13	2329	77.9%	43.9%	3.2	61.8%	48.8%
Moldova	2005	1138	98.4%	93.2%	8.6	100.0%	95.7%
Namibia	2013	1075	95.0%	81.1%	6.1	85.4%	70.8%
Nepal	2011–12	1512	83.7%	50.5%	3.5	38.6%	44.0%
Nigeria	2013	15,118	67.7%	55.6%	5.3	39.9%	45.6%
Rwanda	2015	855	98.9%	43.7%	3.2	92.0%	44.1%
Sierra Leone	2013	2621	97.9%	88.7%	8.1	57.8%	81.6%
Tajikistan	2012	2283	81.4%	57.1%	4.1	79.1%	88.1%
Tanzania	2015–16	5310	98.2%	49.5%	3.6	66.6%	8.0%
The Philippines	2013	4179	95.0%	82.2%	6.4	61.1%	77.9%
Timor-Leste	2009–10	1579	87.1%	55.5%	3.8	20.3%	25.0%
Togo	2013–14	3851	92.8%	55.8%	3.7	72.4%	74.5%
Uganda	2011	1275	95.4%	52.2%	3.7	60.2%	40.4%
Zambia	2013–14	7415	98.6%	54.4%	3.7	71.6%	66.1%
Zimbabwe	2015	3752	94.9%	76.6%	5.1	84.2%	30.4%
*Full sample*	2005–16	166,685	85.8%	57.2%	4.6	62.4%	52.8%

Notes: “Any ANC”, “4+ ANC”, “Facility delivery” and “Postnatal visit” are dichotomous variables. “Postnatal visit” defined as one if the visit occurred within 3 days of birth. Standard deviation for “Total ANC Visits” is 3.6

Focusing first on Table 1, the full sample is constituted by 166,685 women. The smallest county-level sample is drawn from Rwanda (855 women), while the largest country-level sample is drawn from India (25,972 women). The lifetime prevalence of any intimate partner violence is 34.0%, suggesting slightly over a third of women have ever experienced IPV. This corresponds to a lifetime prevalence for emotional IPV of 21.3%, for physical IPV of 26.0%, and for sexual IPV of 8.2%. The estimated lifetime prevalence of any IPV ranges from 9.9% in Comoros to 55.8% in the Democratic Republic of Congo. 

We can also estimate the correlations across different forms of IPV for the full sample. These correlations are 0.50 for emotional and physical IPV; 0.32 for emotional and sexual IPV; and 0.33 for physical and sexual IPV.

In the full sample, 49.3% of women have received only primary education, while 17.8% report any secondary education and 8.0% report any tertiary education. 91.3% of women are currently married, and 36.3% are resident in an urban area. Reported marital status is relatively consistent across the sample, but the share of the population reporting urban status varies significantly (from 17.0% in Malawi to 70.1% in Colombia). The average age of surveyed women in each country sample is generally between 27 and 30, and the mean number of living children ranges between 1.5 and 5. The final column of Table 1 reports the mean wealth quintile in each country sample.

Table 2 reports descriptive statistics for the outcome variables of interest, utilization of reproductive health care. In the pooled sample, 85.8% of women report receiving antenatal care, though only 57.2% report receiving the recommended four or more antenatal visits; the average number of visits reported is 4.6. In fact, a majority of women report attendance at antenatal care in all country samples, though the percentage of women reporting attendance ranges from 56.8% in Afghanistan to 99.3% in Jordan.

62.4% of women report that their delivery was in a health facility, and 52.8% reported that they received postnatal care. The lowest rate of facility delivery is observed in Liberia at 0%, and the highest rate observed in Moldova (100%). Thus in general, there is meaningful variation in utilization of reproductive health care across the sample. The lowest rates are generally observed in sub-Saharan Africa and in Afghanistan, while the highest rates are observed in Jordan and in the three countries in the sample drawn from Europe and Central Asia (Moldova, the Kyrgyz Republic and Tajikistan).

### Relationship between IPV and utilization of reproductive health care

Table 3 reports the results analyzing any intimate partner violence as an explanatory variable. Again, we present three models: unadjusted (Model 1), adjusted for country of residence (Model 2), and adjusted for country of residence, subnational region of residence, and sociodemographic characteristics (Model 3). Note that when models two and three are estimated, clusters (as defined by country or subnational region) in which there is no variation in the dependent variable are dropped, generating minor variation in sample size.

**Table 3:** Any IPV as Risk Factor for Care Utilization

Variables	Any ANC visit			4+ ANC visits			Total ANC visits			Facility delivery			Postnatal visit		
	OR	95% CI	*p*-value	OR	95% CI	p-value	Coefficient estimate	95% CI	p-value	OR	95% CI	p-value	OR	95% CI	p-value
**Model 1 (unadjusted)**															
Any IPV	0.85	0.58,1.25	0.406	0.76	0.59,0.97	0.026	− 0.56	−0.98,-0.14	0.011	0.87	0.73,1.04	0.125	0.77	0.65,0.91	0.002
Observations	166,685			166,685			166,685			166,685			166,685		
**Model 2 (adjusted for country of residence)**															
Any IPV	0.91	0.68,1.22	0.523	0.80	0.67,0.96	0.018	− 0.27	− 0.58,0.04	0.088	0.82	0.68,0.98	0.030	0.89	0.73,1.07	0.222
Observations	166,685			166,685			166,685			164,022			166,685		
**Model 3 (adjusted for country of residence, subnational region of residence, and sociodemographic characteristics)**															
Any IPV	0.96	0.89,1.04	0.329	0.89	0.85,0.94	0.000	−0.09	−0.15,-0.02	0.009	0.95	0.90,1.00	0.030	0.97	0.94,1.01	0.176
Observations	163,453			166,060			166,685			163,014			166,039		

*Notes*: *OR* odds ratio, *CI* confidence interval, *ANC* antenatal care. “Any IPV” is any physical or sexual violence by partner in lifetime. “Postnatal visit” is postnatal visit for mother or newborn within 3 days. All Models use heteroskedasticity-robust standard errors clustered at the country level. Model 1 does not include any controls. Model 2 controls for dichotomous variables for each country. Model 3 controls for dichotomous variables for each country, subnational region, dichotomous variables for the respondent’s age in years, dichotomous variables for the respondent completing primary, secondary or tertiary education, a dichotomous variable for current marital status, total number of children born, dichotomous variables for wealth quintile, and a dichotomous variable for urban residence

In Model 1, no significant association is observed between lifetime experience of any IPV and utilization of antenatal care (OR = 0.85; 95% CI: 0.58, 1.25; *p* = 0.406) or facility delivery (OR = 0.87; 95% CI: 0.73, 1.04; *p* = 0.125). However, there is a significant association between lifetime experience of any IPV and utilization of four or more antenatal care visits (OR = 0.76; 95% CI: 0.59, 0.97; *p* = 0.026), and utilization of postnatal care (OR = 0.77; 95% CI: 0.65, 0.91; *p* = 0.002). In addition, there is also a significant and negative linear relationship between lifetime experience of any IPV and the number of ANC visits. These results are generally consistent in Model 2; however, the relationship between lifetime experience of any IPV and facility delivery is now statistically significant, while the relationship between lifetime experience and postnatal care is no longer statistically significant.

In Model 3, the model fully adjusting for sociodemographic characteristics, no significant association is observed between lifetime experience of any IPV and utilization of any antenatal care (AOR = 0.96; 95% CI 0.89, 1.04; *p* = 0.329), or between lifetime experience of any IPV and attendance at postnatal care (AOR = 0.97; 95% CI: 0.94, 1.01; *p* = 0.176). However, there is a significant association between lifetime experience of any IPV and utilization of four or more antenatal care visits (AOR = 0.89; 95% CI: 0.85, 0.94; *p* = 0.000) and facility delivery (AOR = 0.95; 95% CI: 0.90, 1.00; *p* = 0.030). In addition, there is also a significant and negative relationship between lifetime experience of any IPV and the number of antenatal care visits (estimated coefficient − 0.09; 95% CI -0.15, − 0.02; *p* = 0.009).

Table 4 reports the results for the same three models, now estimated using separate dichotomous variables for emotional violence, physical violence and sexual violence as explanatory variables; given the increased number of coefficients, we highlight only those that are statistically significant in the text. In Model 1, there is a positive and significant association between lifetime experience of emotional IPV and any use of antenatal care (OR = 1.12; 95% CI: 1.00, 1.26; *p* = 0.050), and a negative and significant association between physical IPV and the utilization of four or more antenatal care visits (OR = 0.69; 95% CI: 0.49, 0.96; *p* = 0.027) as well as the utilization of postnatal care (OR = 0.77; 95% CI: 0.63, 0.95; *p* = 0.014). There is also a negative and significant association between sexual IPV and the utilization of postnatal care (OR = 0.77; 95% CI: 0.61, 0.95; *p* = 0.016). In addition, there are significant and negative relationships between experience of physical IPV and experience of sexual IPV and the number of antenatal care visits (estimated coefficient for physical IPV -0.65; 95% CI: − 1.28, − 0.03; *p* = 0.042; estimated coefficient for sexual IPV -0.52, 95% CI: − 1.02, − 0.01; *p* = 0.046).

**Table 4:** Emotional, Physical, and Sexual Violence as Risk Factors for Care Utilization

Variables	Any ANC visit			4+ ANC visits			Total ANC visits			Facility delivery			Postnatal visit		
	OR	95% CI	*p*-value	OR	95% CI	*p*-value	Coefficient estimate	95% CI	*p*-value	OR	95% CI	*p*-value	OR	95% CI	*p*-value
**Model 1 (unadjusted)**															
Emotional violence	1.12	1.00,1.26	0.050	1.09	0.92,1.28	0.325	0.12	−0.14,0.38	0.342	1.13	0.92,1.39	0.239	1.03	0.88,1.20	0.713
Physical violence	0.72	0.44,1.16	0.172	0.69	0.49,0.96	0.027	−0.65	−1.28,-0.03	0.042	0.80	0.61,1.06	0.119	0.77	0.63,0.95	0.014
Sexual violence	1.03	0.82,1.28	0.826	0.84	0.65,1.09	0.187	−0.52	−1.02,-0.01	0.046	0.87	0.72,1.05	0.152	0.77	0.61,0.95	0.016
Observations	166,685			166,685			166,685			166,685			166,685		
**Model 2 (adjusted for country of residence)**															
Emotional violence	1.13	0.96,1.33	0.149	0.99	0.92,1.06	0.719	0.02	−0.06,0.09	0.661	1.06	0.97,1.15	0.192	1.04	0.98,1.10	0.213
Physical violence	0.84	0.68,1.03	0.093	0.81	0.69,0.94	0.005	−0.24	− 0.57,0.09	0.149	0.79	0.68,0.92	0.002	0.89	0.74,1.07	0.218
Sexual violence	0.86	0.79,0.95	0.003	0.86	0.78,0.95	0.003	−0.33	−0.57,-0.09	0.008	0.82	0.76,0.89	0.000	0.84	0.79,0.90	0.000
Observations	166,685			166,685			166,685			164,022			166,685		
**Model 3 (adjusted for country of residence, subnational region of residence, and sociodemographic characteristics)**															
Emotional violence	1.00	0.93,1.07	0.963	0.96	0.91,1.01	0.111	0.00	−0.04,0.04	0.926	1.04	0.98,1.10	0.162	1.01	0.96,1.06	0.628
Physical violence	0.92	0.89,0.96	0.000	0.89	0.85,0.94	0.000	−0.10	−0.19,-0.02	0.018	0.91	0.86,0.95	0.000	0.96	0.90,1.02	0.179
Sexual violence	1.03	0.92,1.15	0.625	0.98	0.92,1.04	0.498	−0.05	−0.13,0.03	0.185	0.99	0.92,1.07	0.863	0.99	0.90,1.08	0.815
Observations	163,453			166,060			166,685			163,014			166,039		

Notes: *OR* odds ratio, *CI* confidence interval, *ANC* antenatal care. “Emotional violence” is a dichotomous variable for emotional violence by partner in lifetime. “Physical violence” is a dichotomous variable for physical by partner in lifetime. “Sexual violence” is a dichotomous variable for sexual violence by partner in lifetime. “Postnatal visit” is postnatal visit for mother or newborn within 3 days. All Models use heteroskedasticity-robust standard errors clustered at the country level. Model 1 does not include any controls. Model 2 controls for dichotomous variables for each country. Model 3 controls for dichotomous variables for each country, subnational region, dichotomous variables for the respondent’s age in years, dichotomous variables for the respondent completing primary, secondary or tertiary education, a dichotomous variable for current marital status, total number of children born, dichotomous variables for wealth quintile, and a dichotomous variable for urban residence

In Model 2, the results become considerably more precise. There are now significant and negative associations between experience of physical IPV and utilization of any antenatal care (AOR = 0.84; 95% CI: 0.68, 1.03; *p* = 0.093), utilization of four or more antenatal care visits (AOR = 0.81; 95% CI: 0.69, 0.94; *p* = 0.005) and facility delivery (AOR = 0.79; 95% CI: 0.68, 0.92; *p* = 0.002). There are also negative associations between the experience of sexual IPV and utilization of any antenatal care (AOR = 0.86; 95% CI: 0.79, 0.95; *p* = 0.003), four or more antenatal care visits (AOR = 0.86; 95% CI: 0.78, 0.95; p = 0.003), facility delivery (AOR 0.82; 95% CI: 0.76, 0.89; *p* = 0.000), and postnatal care (AOR = 0.84, 95% CI: 0.79, 0.90; p = 0.000). The linear relationship between experience of sexual IPV and total ANC visits is also statistically significant (estimated coefficient − 0.33, 95% CI: − 0.57, − 0.09; *p* = 0.008).

In Model 3, there is a negative and significant association between lifetime experience of physical IPV and utilization of any antenatal care (AOR = 0.92; 95% CI: 0.89, 0.96; p = 0.000), more than four antenatal care visits (AOR = 0.89; 95% CI: 0.85, 0.94; p = 0.000), and facility delivery (AOR = 0.91; 95% CI: 0.86, 0.95; p = 0.000). There is also a significant and negative relationship between lifetime experience of physical IPV and the number of antenatal care visits (estimated coefficient − 0.10, 95% CI: − 0.19, − 0.02; *p* = 0.018). In this model, there are no significant associations between experience of emotional and sexual IPV and any measures of health services utilization, and there is no significant association between experience of any form of IPV and postnatal care utilization.

Table 5 reports the results estimated using a dichotomous variable for any IPV, but splitting the sample over three time periods; for concision, only results estimating Model 3 are reported. In the earliest time period (2005—2009), experience of any IPV is significantly associated with utilization of any antenatal care (AOR = 0.95, 95% CI: 0.91, 0.98; *p* = 0.004), more than four antenatal care visits (AOR = 0.88, 95% CI: 0.84, 0.94; *p* = 0.000), and facility delivery (AOR = 0.93; 95% CI 0.91, 0.96; p = 0.000). There is also a significant and negative relationship between lifetime experience of physical IPV and the number of antenatal care visits (estimated coefficient − 0.16, 95% CI: − 0.19, − 0.13; *p* = 0.001). In the later time periods, these associations are generally more noisily estimated and some are statistically insignificant. In the period 2010—2014, experience of any IPV is significantly associated only with utilization of more than four antenatal care visits (AOR = 0.91, 95% CI: 0.86, 0.96; p = 0.001), and in the period 2015—2016, experience of any IPV is significantly associated with utilization of any antenatal care (AOR = 0.92; 95% CI: 0.87, 0.97; *p* = 0.002).

**Table 5:** Any IPV as Risk Factor for Care Utilization, Heterogeneity by Survey Year

Variables	Any ANC visit			4+ ANC visits			Total ANC visits			Facility delivery			Postnatal visit		
	OR	95% CI	*p*-value	OR	95% CI	*p*-value	Coefficient estimate	95% CI	*p*-value	OR	95% CI	*p*-value	OR	95% CI	*p*-value
**Survey years 2005–2009; Model 3**															
Any IPV	0.95	0.91,0.98	0.004	0.88	0.84,0.94	0.000	−0.16	− 0.19,-0.13	0.001	0.93	0.91,0.96	0.000	0.98	0.95,1.01	0.227
Observations	29,415			29,931			29,931			27,268			29,931		
**Survey years 2010–2014; Model 3**															
Any IPV	0.99	0.87,1.12	0.829	0.91	0.86,0.96	0.001	−0.05	−0.11,0.02	0.183	0.96	0.90,1.02	0.192	0.98	0.93,1.03	0.357
Observations	93,555			95,178			95,185			94,124			94,519		
**Survey years 2015–2016; Model 3**															
Any IPV	0.92	0.87,0.97	0.002	0.90	0.78,1.03	0.129	−0.09	−0.21,0.02	0.091	0.95	0.86,1.05	0.331	0.97	0.91,1.04	0.389
Observations	40,446			40,935			41,569			41,558			41,564		

*Notes*: *OR* odds ratio, *CI* confidence interval, *ANC* antenatal care. “Any IPV” is any physical or sexual violence by partner in lifetime. “Postnatal visit” is postnatal visit for mother or newborn within 3 days. All regressions estimate Model 3, controlling for dichotomous variables for each country, subnational region, dichotomous variables for the respondent’s age in years, dichotomous variables for the respondent completing primary, secondary or tertiary education, a dichotomous variable for current marital status, total number of children born, dichotomous variables for wealth quintile, and a dichotomous variable for urban residence

## Discussion

Our primary results suggest that experiencing any IPV is a risk factor for decreased use of maternal health services in a broad sample of births observed in lower and middle-income countries. Although lifetime experience of any IPV was not significantly associated with attending at least one ANC visit, a utilization measure attained by more than 85% of our study sample, it was significantly and negatively associated with three other measures of health services utilization (utilization of four or more ANC visits, number of ANC visits, and facility delivery), and this association remained statistically significant even after adjusting for country of residence, subnational region of residence, and additional individual-level covariates. The association between intimate partner violence and postnatal care is weak, however; in the adjusted models (Models 2 and 3), this association was statistically insignificant.

When we examine the relative roles of different types of IPV, in general the association between physical IPV and health services utilization is the most robust. In the model fully adjusting for region of residence and individual-level covariates, only physical IPV is significantly associated with health services utilization. The magnitude of the observed associations ranges from 0.89 to 0.92 (Model 3). Again, there is no significant association between physical IPV and use of postnatal care. As previously reported, there are positive correlations between experience of physical IPV and experience of both emotional and sexual IPV (of magnitude approximately .5 and .3 respectively). The estimated results thus suggest that women who are experiencing emotional and sexual IPV may be less likely to utilize reproductive healthcare if they are also experiencing physical IPV, but that there is no detectable shift for women experiencing only emotional or sexual IPV.

Two outcome variables examined in this analysis (any utilization of antenatal care, and attendance at four or more antenatal care visits) are broadly consistent with variables examined in the existing literature [14, 16, 19, 20, 22, 23, 25]. However, some analyses set the cutoff for sufficient ANC at three or five visits, rather than four. In addition, previous papers generally analyze skilled attendance at delivery [14, 19, 20, 23, 25], while this analysis focuses on facility-based delivery; two previous papers analyzed delivery at home or outside the facility, the inverse of the measure employed in this analysis [16, 21]. The analysis of facility-based delivery as a key indicator of maternal health services utilization is consistent with emerging evidence that provision of skilled delivery care in facilities is more efficient and less costly when compared to provision of skilled care at home, particularly as demand for delivery care rises [31, 32], and that provision of skilled delivery care in communities can be challenging due to the absence of required supplies, limited access to an appropriate delivery environment, and limited access to referrals [33, 34]. This is the second paper to analyze the relationship between experience of IPV and the number of ANC visits, as well as postnatal care, following a recent analysis drawing on data from Uttar Pradesh in India [35].

Relative to the existing literature, our findings are consistent with a number of papers that have used data drawn from a single country and found a negative association between IPV and antenatal care utilization in Bangladesh [19, 23], Ethiopia [17], Ghana [22], Honduras [15], India [24], and Timor-Leste [16]. Our findings are similarly consistent with a number of single-country studies that have found a negative association between IPV and skilled attendance at delivery (or facility-based delivery) in Bangladesh [19, 21, 23], Ethiopia [17], Kenya [14], Timor-Leste [16], and Uganda [25], and in a six-country sample encompassing Bangladesh, Cambodia, Colombia, Egypt, the Ukraine and Zambia [20]. A recent systematic review similarly documented an overall pattern in which women who have experienced IPV demonstrate reduced utilization of antenatal care and skilled delivery care [4]. One relatively novel finding in our analysis suggests that the association between IPV and health services utilization may be weakening over time and seems largest in the 2000—2005 period; however, this finding should be interpreted cautiously, given that the sample size for each time period is reduced, and power may be limited.

Our findings are also consistent with a large literature documenting substantial experience of IPV during pregnancy for women in developing countries. A number of systematic reviews have documented prevalence rates of IPV in LMICs ranging from 2% up to 65%, depending on the setting and the type of violence [36–39]. While it is challenging to generalize about the relative prevalence of IPV during pregnancy, one systematic review focused on sub-Saharan Africa concluded that the prevalence of IPV was roughly constant for pregnant and non-pregnant women [37], and a second noted that the relationship between the prevalence of IPV for women of reproductive age and the prevalence during pregnancy is context-dependent [38]. It is clear, however, that IPV during pregnancy is by no means a rare phenomenon, and this is consistent with the observed relationship between reported lifetime experience of IPV and utilization of maternal health services.

When comparing this analysis to the existing literature, the associations observed between IPV and various measures of care utilization estimated in this analysis are generally somewhat reduced in magnitude. For example, a recent meta-analysis estimates that women who experienced IPV had 25% reduced odds of accessing adequate antenatal care (AOR = 0.75) and 20% reduced odds of utilizing skilled delivery care for women experiencing IPV (AOR = 0.80) [4]. Analysis of a six-country sample similarly reports women who experienced IPV had 24% reduced odds in the probability of skilled assistance at birth (AOR = 0.76) [20]. Other single-country analyses report similar estimates. The estimates in this paper, however, suggest that in a large sample of women from LMICs, women who experience IPV have around a 5–10% reduction in the odds of accessing reproductive health care. In light of this finding, it is important to note that the utilization of maternal health care can be shaped by a wide range of factors beyond IPV, including socioeconomic status, community norms, supply-side factors reflecting the availability and organization of health care services, and past health history; these factors have been explored in a number of systematic reviews analyzing utilization of health services [3, 40–42]. The importance of these additional determinants may be one reason that the observed association between IPV and services utilization, while precisely estimated, is not large in magnitude.

Importantly, there is little previous evidence in the literature around the relationship between experience of IPV and utilization of postnatal care. Two papers analyze the associations between experience of IPV and postnatal care practices and postnatal care receipt (kangaroo mother care, initiation and continuation of breastfeeding, and post-partum contraceptive use) in India, and find a heterogeneous pattern. There is evidence that experience of physical and sexual IPV reduces early initiation of breastfeeding and exclusive breastfeeding [13], as well as reducing the number of health topics discussed in postnatal visits [35]. Our paper is the first to examine the relationship between experience of IPV and utilization of postnatal care from a skilled provider in a large sample, and we find no evidence of a significant association. Utilization of postnatal care in our sample is considerably lower (52.8%) vis-à-vis utilization of antenatal care (85.8%) and skilled attendance at delivery (62.4%), but there is no evidence that experience of IPV increases the risk of failing to access postnatal care.

This evidence also highlights the importance of further examining the association between experience of IPV and maternal and neonatal health outcomes in developing country contexts. In the literature to date, two overview papers summarize a wide variety of evidence linking IPV to pregnancy and birth outcomes, but the majority of this evidence is drawn from developed countries [9, 43]. More recent evidence from Bangladesh finds a significant association between experience of IPV and pregnancy loss through miscarriage, induced abortion or stillbirth [44], though a paper analyzing IPV during pregnancy in Delhi found this relationship was statistically insignificant [45]. Another study analyzing data from Tamil Nadu and Uttar Pradhesh found a significant association between wife-beating and fetal and infant death [46], and there is also evidence of significant associations between physical IPV and pregnancy termination in Timor-Leste and Guatemala City [47, 48], and between any IPV and neonatal and infant mortality in a multicountry sample in East Africa [49]. In addition, three smaller-scale studies in Mexico, Nicaragua and South Africa report statistically significant associations between physical abuse during pregnancy and low birth weight [50–52]. To our knowledge, however, no systematic cross-regional study has examined these relationships.

Our findings raise two key questions of interpretation: first, why is the association between experience of physical IPV and health services utilization more robust than the association between emotional and sexual IPV and the same utilization measures; and second, why is the association between experience of physical IPV and postnatal care statistically insignificant? On the first question, only three two other papers in the existing literature examined the relationship between all forms of IPV and care utilization. In Kenya, only emotional IPV is significantly associated with utilization of skilled delivery care [14], while in Bangladesh, only physical IPV is significantly associated with utilization of antenatal care [23]; evidence from Ethiopia suggests that all three forms of IPV are associated with different variables capturing care utilization [17]. One plausible hypothesis for the significant association between physical IPV and care utilization is linked to reporting patterns: reporting of physical IPV may be more socially acceptable vis-à-vis reporting of other forms of violence, given that physical IPV is more visible. If other forms of violence are systematically underreported, this could attenuate any correlation between these other forms of IPV and care utilization. Alternatively, women experiencing physical IPV may be more likely to be explicitly prohibited from using health services by the perpetrator.

On the second point, unpacking the null relationship between physical IPV and utilization of postnatal care, it is possible that the absence of a significant relationship reflects the fact that women’s experience of IPV itself is reduced in the immediate postpartum period. Evidence around this point from developing countries is very limited. A summary review paper reporting evidence around IPV during pregnancy in developing countries does not report any separate estimates for pregnancy vis-à-vis the postpartum period [39]. One paper analysing IPV in Saudi Arabia states that the risk of IPV is higher in the postpartum period [53], but presents no quantitative evidence, and one paper analysing IPV in India finds that reported experience of IPV is somewhat lower in the post-partum period (17.5%) compared to reported experience during pregnancy (24.4%) [54]. However, this evidence must be considered tentative. Another hypothesis is that the quality of care experienced during the delivery itself, as well as any experience of obstetric maltreatment --- a phenomenon increasingly documented in recent literature --- is a key determinant of utilization of postpartum care, thus rendering any experience of IPV less salient [55].

This study had several key strengths and limitations. One major strength is the scope of the sample: we use data from over 167,000 women in 36 countries. By contrast, the largest sample previously employed in a parallel analysis included only 18,507 women in six countries [20]. A second strength is that we present evidence on all of the key steps in the maternal health “cascade” (e.g., antenatal services, delivery services, and post-natal services) and measure at least one of these outcomes on both the extensive margin (“Any ANC visit”) and the intensive margin (“Total ANC visits”). This reveals that IPV is not a risk factor for ANC attendance on the extensive margin, yet is a key risk factor for attendance on the intensive margin. In addition, we analyse associations between all forms of intimate partner violence (emotional, physical, sexual and any IPV) and utilization of health services, and demonstrate that the primary results are robust to adjusting for country and subregion of residence as well as demographic controls. No previous paper analysed the relationship between IPV and all forms of maternal health services utilization, including postnatal care utilization, and only three previous papers analysed the relationship between all dimensions of intimate partner violence and health services utilization, in Bangladesh [23], Ethiopia [17], and Kenya [14].

The key limitations of this study are as follows. First, the DHS measures self-reported IPV, and this variable may undercount IPV among respondents in our study sample. Challenges with self reporting may be particularly acute in a long multitopic health survey such as the DHS, as distinct from a survey focusing specifically on IPV. This would serve to attenuate our estimated odds ratios (and regression coefficients) toward zero. In addition, we use a measure of lifetime experience of IPV to capture exposure to violence, but the point at which violence was experienced is not specified and may not overlap with the time period leading up to the birth for which care utilization is reported. However, it should be noted that this strategy of examining associations between lifetime experience of IPV and care utilization is widely used in the literature [13, 14, 19–21]. Second, given the large scale of the sample, we are not able to systematically characterize the organization of maternal health services in the 36 countries comprising the sample, identify supply-side constraints in services, and describe how these constraints may affect utilization patterns. Third, although the geographic scope of our sample is large, IPV modules are only included in the standard DHS for fewer than forty countries, somewhat limiting the global scope of our analysis.

Our findings have implications for both future programming and research. Given evidence that experience of IPV reduces utilization of maternal health care, interventions targeting increased utilization of maternal health care in developing country contexts may benefit from identifying women experiencing IPV and specifically targeting barriers linked to IPV. Future research in this area may also qualitatively explore the channels that lead women who have experienced IPV to demonstrate a reduced propensity to utilize maternal health care; some potential channels include the inability to obtain a male partner’s permission to visit a facility, lack of access to financial or logistical support, and fear of retaliation from a male partner. In addition, given that there is no evidence of a significant association between experience of IPV and utilization of postnatal care, future work could usefully explore whether the effect of these channels is attenuated in the immediate postpartum period.

Another area relevant for future research is the identification of channels through which physical IPV has a significant effect on utilization of IPV, while emotional and sexual IPV does not. Based on the patterns observed in this analysis, we may hypothesize that emotional violence does not exert a significant influence on maternal health decision-making. On the other hand, sexual violence may increase women’s anxiety around utilizing care while simultaneously increasing the risk of health complications requiring care during pregnancy, given the evidence that women experiencing forced sex or sexual IPV are at particularly high risk of reproductive health challenges [6]. The net effect of two shifts, one associated with more utilization of care and one associated with less utilization of care, could yield an overall null effect.

However, these channels require further exploration using both qualitative and quantitative data. The role of perpetrators in directly limiting women’s access to health services would also benefit from further exploration, particularly in light of the observed positive correlation between different forms of IPV. Men who perpetrate physical IPV are also more likely to perpetrate other forms of IPV, but further work is needed to elucidate why the former type of violence differentially affects health seeking behavior for the women who experience violence.

## Conclusions

This paper provides novel evidence of a significant association between lifetime experience of physical intimate partner violence and utilization of maternal health services, utilizing a novel large-scale dataset including more than 166,000 women in 36 low and middle-income countries. We present evidence that lifetime experience of physical IPV is associated with a reduction in the use of any antenatal care and the intensity of antenatal care use, and a reduction in the utilization of facility care at birth. However, there is no evidence of any relationship between experience of IPV and utilization of postnatal care, and no evidence of any relationship between experience of sexual or emotional IPV and utilization of any form of maternal health services. This is the first paper to identify the relationship between experience of IPV and care utilization in a large-scale dataset, highlighting that this relationship may be an important channel for adverse health effects of IPV for women of reproductive age.

## Data Availability

The data analyzed in this article is publicly available from the Demographic and Health Surveys program (www.dhsprogram.com).
